# Improving Compliance with the CMS SEP-1 Sepsis Bundle at a Community-Based Teaching Hospital Emergency Department

**DOI:** 10.51894/001c.37707

**Published:** 2022-09-06

**Authors:** Marius Alexander, Melissa Sydney, Ari Gotlib, Megan Knuth, Olga Santiago-Rivera, Nikolai Butki

**Affiliations:** 1 Emergency Medicine Residency, McLaren Health Care/Oakland/MSU; Michigan State University College of Osteopathic Medicine; 2 Osteopathic Medical Specialties Michigan State University College of Osteopathic Medicine; 3 Michigan State University College of Osteopathic Medicine; Graduate Medical Education, McLaren Health Care/Oakland/MSU; 4 Emergency Medicine Residency, McLaren Health Care/Oakland/MSU; Michigan State University College of Osteopathic Medicine; Graduate Medical Education, McLaren Health Care/Oakland/MSU

**Keywords:** Sepsis, bundle, emergency department, quality improvement, patient safety

## Abstract

**INTRODUCTION:**

The Centers for Medicare & Medicaid Services (CMS) designed Hospital Quality Initiatives (HQI) to assure delivery of quality health care for institutions receiving Medicare payments. Like many teaching institutions, the SEP-1 compliance rates at McLaren Oakland in Pontiac fluctuated monthly and were not achieving institutional target expectations.

**METHODS:**

The project team designed a Sepsis Macro and a Sepsis Order Set in the electronic medical record system. The project team also implemented an educational initiative targeted at emergency medicine resident and attending physicians. The educational initiative instructed emergency medicine resident and attending physicians in the metrics measured in the SEP-1 bundle as well as how to properly use the newly designed Sepsis Macro and Sepsis Order Set.

**RESULTS:**

After implementation of the Sepsis Macro and Sepsis Order Set, the overall compliance with the SEP-1 bundle improved from 57% to 62%, above national averages and at the institutional target expectations. However, there were not statistically significant differences (p = 0.562) between the compliance rate before and after program implementation (Pre = 57% (SD = 0.27); 95% CI: 0.29 - 0.85); Post= 62% (SD = 0.11); 95% CI: 0.55 - 0.70). After program implementation the SEP-1 compliance rate was met in 82% of the months in comparison with 50% of the months in the pre-intervention (p = 0.28).

**CONCLUSIONS:**

Although not achieving statistical significance, this intervention demonstrated that simple, cost-effective measures of education and standardization in documentation and order entry in EMR’s can improve clinically significant compliance to CMS HQI metrics in community-based teaching institutions.

## INTRODUCTION

The Centers for Medicare & Medicaid Services (CMS) designs Hospital Quality Initiatives (HQI) to assure delivery of quality health care for institutions receiving Medicare payments.^1^ One HQI that CMS has measured since October 2015 is the “Early Management Bundle, Severe Sepsis/Septic Shock Measure”, commonly referred to as SEP-1.^2,3^ The measure’s target population is adult inpatients 18 years and older with a diagnosis of severe sepsis or septic shock.

Sepsis is defined as having a source of infection plus two or more systemic inflammatory response syndrome (SIRS) criteria: Temperature >38^0^ or < 36^0^ Celsius, heart rate > 90, respiratory rate >20 or PaCO2<32mm Hg, white blood cell count > 12,000/mm^3^ or <4,000mm^3^ or >10% bands. Severe sepsis is defined as sepsis plus organ dysfunction: Serum lactic acid above upper limit of normal or systolic blood pressure <90mm Hg or a drop of > 40mm Hg of normal. Septic shock is defined as severe sepsis with hypotension despite adequate fluid resuscitation.^4^ Studies have shown that hospitals demonstrating compliance with SEP-1 have superior process measures (e.g., serum lactate measurement)^5^ or have positive patient outcomes including lower mortality, length of stay and readmission rates.^6^

To be deemed compliant with SEP-1, a healthcare facility must demonstrate compliance with all the metrics in each category. The term ‘bundle’ is used to refer to the grouping of all the metrics measured. There are two bundles included in the SEP-1 measure: the severe sepsis bundle and the septic shock bundle. The severe sepsis bundle requires lactate measurements, blood cultures and broad-spectrum antibiotics administration within three hours of sepsis identification followed by repeat lactate measurements within six hours if the initial lactate level is elevated. The septic shock bundle adds three additional requirements: 1. 30 mL/kg of IV fluids within three hours; 2. vasopressors within five hours for persistent hypertension; and 3. repeat volume assessment within six hours. The SEP-1 core measure dictates that all interventions must be attained for a case to be deemed compliant with the measure. This is known as an “all-or-nothing” requirement that is particularly challenging for hospitals and providers to demonstrate compliance.^7^

Like many other institutions, McLaren Oakland SEP-1 compliance rates have historically fluctuated month by month and during the last months of 2020 were not demonstrating meeting all criteria to be determined compliant with the measure. Acknowledging that both arms of the SEP-1 bundle originate in the emergency department (ED), a multidisciplinary team led by ED physicians launched a sepsis quality improvement/patient safety project to increase institutional compliance with the SEP-1 bundle. Previous studies have found improvement in individual components of the SEP-1 or in the SEP-1 compliance bundle when ED is involved in quality improvement projects.^8–10^

## METHODS

### Project Purpose

The aim of this project was to improve compliance with sepsis CMS HQI metrics for adult patients admitted to McLaren Oakland, a community-based university-affiliated teaching hospital with approximately 31,000 ED visits per year and 4,500 admissions per year. The team for the SEP-1 project included two ED attending physicians (AG, NB), two ED resident physicians (MA, MS), one staff from the Quality Department, one rotating medical student (MK) and the Graduate Medical Education Advisor for Scholarly Activities (OSR).

For the needs assessment, the team initiated a cause-and-effect discussion using an Ishikawa, or ‘fishbone’ Diagram,^11^ identifying two causal factors most likely accounting for the low compliance with the SEP-1 bundle. The first was a lack of working knowledge among ED physicians of the specific metrics in the SEP-1 bundle. The second was inconsistent ordering of the requisite orders for the SEP-1 bundles and documentation of care provided to patients with sepsis in the electronic medical record (EMR) by the ED physicians. The team developed a project protocol that was reviewed by the Institutional IRB which was determined to be non-human subject research.

### Study Intervention

**Figure 1.**
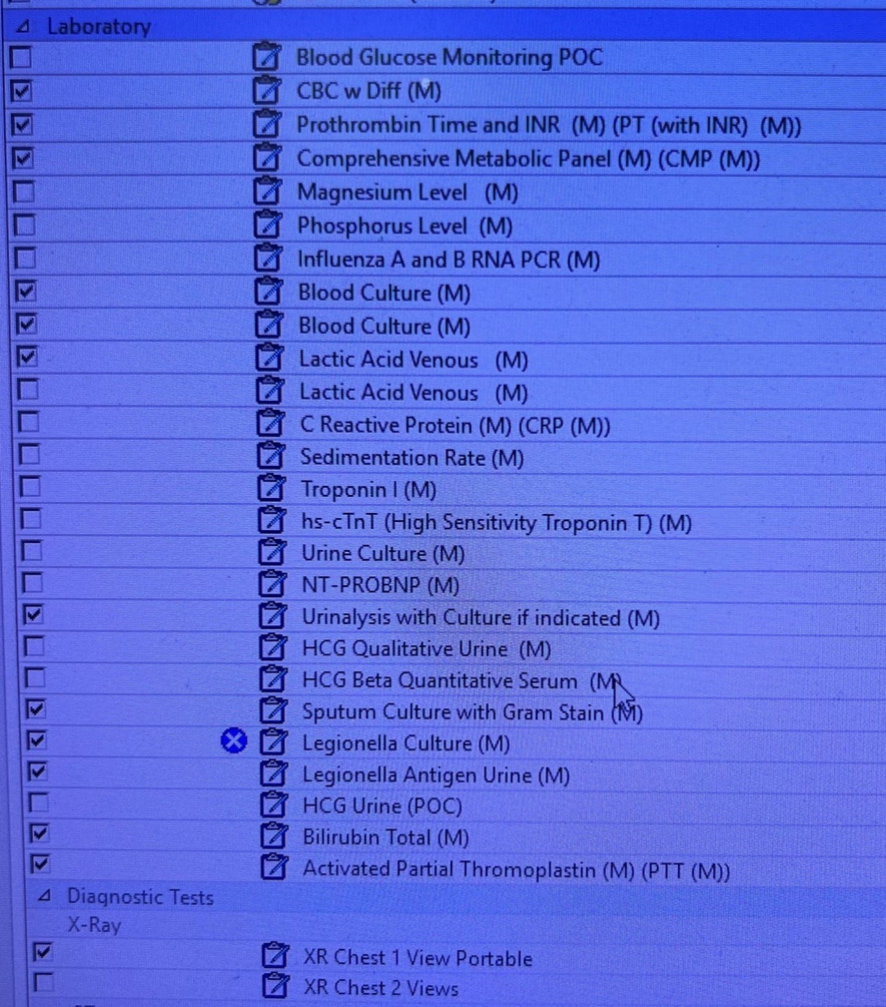
Sepsis Order-Set

**Figure 2.**
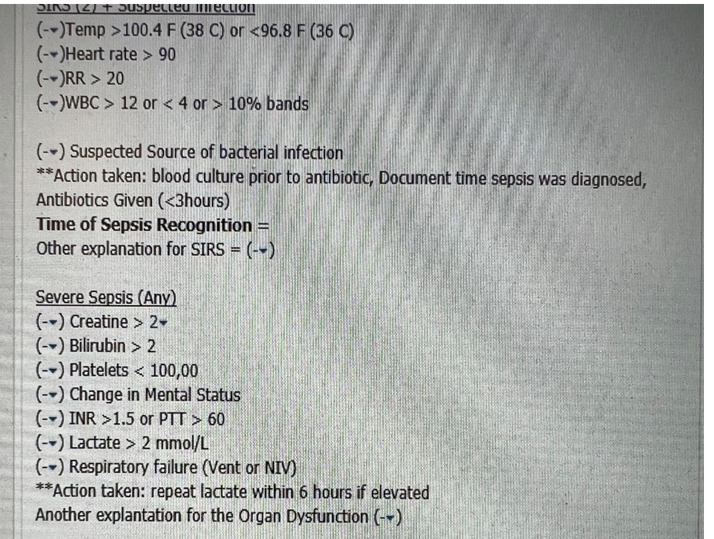
Sepsis Macro

To improve the compliance rate for SEP-1, the project targeted ED resident and attending physicians. The project consisted of two components: The first was improving the processes of ordering the requisite laboratory studies and physician documentation in the EMR. To achieve these, two forms in the EMR were developed: (1) a Sepsis Order-Set (Figure 1) to assure all requisite orders to comply with the SEP-1 bundle are ordered; and (2) a Sepsis Macro (Figure 2) for documentation in the EMR.

A benefit of the EMR used by the institution is that the EMR allows individual providers to design unique macros and order sets as time-saving devices tailored to each provider’s practice pattern. A macro, colloquially referred to as a ‘dot-phrase’, is a time-saving predefined phrase a provider can summon when documenting a note in the EMR.

This allowed ED physicians to quickly summon a macro as a template for the physician to input the requisite data to demonstrate compliance into their documentation when treating a patient with sepsis. The provider individuality of this function, however, can lead to variation in order entry and documentation between providers. To standardize the practice of sepsis management among the ED physicians to comply with the SEP-1 bundle, the project team designed both a Sepsis Macro and Sepsis Order Set.

The Sepsis Macro was designed in coordination between the ED providers and the EMR software staff. The Sepsis Macro also includes reminders on specific tasks that need to be completed to be deemed compliant with the SEP-1 bundle. These include repeating a second lactate if the initial lactate was elevated and ordering a 30cc/kg fluid bolus if the lactate is above a certain number. The Sepsis Order Set was also designed in coordination with the EMR software staff to include a pre-populated checklist to include all the requisite orders needed to comply with the SEP-1 bundle.

The second component of the project was training the ED physicians on the SEP-1 metrics and on the Sepsis Macro and Sepsis Order Set. In April 2021 the project leaders delivered the educational intervention, a one-hour session training 18 ED resident and seven attending physicians on the metrics measured in the SEP-1 bundle and how to use both the newly designed Sepsis Macro and Sepsis Order Set.

The educational intervention also included monthly chart reviews of cases that qualified for sepsis reporting. From May 2021- June 2021 between three and five cases were discussed each month with the ED resident and attending physicians during the monthly emergency medicine operations meeting. Cases that were compliant with the SEP-1 bundle the prior month were reviewed as exemplar practices to model behavior. Cases that were deemed noncompliant, identifying the specific reasons why the case ‘fell out’ of compliance, were also reviewed.

### Study Measures

The population measured in the project included all adult patients that presented to the ED during the study period with a diagnosis of sepsis. The compliance rate with the SEP-1 was determined by the institutional data extractor trained in data abstraction specific to CMS HQI metrics. The data analyzed is the de-identified data the institution reported to CMS. The reported SEP-1 compliance measured by the compliance department and reported to CMS were compared prior to and post project implementation. We created two dichotomous variables to categorize the periods before and after program implementation (0 = Nov 2020 to April; and 1 = June 2021 to April 2022; and if the target compliance rate was met (0 = Not met; 1 = Met). Target goals for the sepsis quality measures for each institution change at the beginning of the fiscal year (October). The reason for annual reassessment and changes in target goals is make it more possible for the hospitals to achieve a better score and improve the results and to strive for better scores from the previous year.

The data analyst (OSR) used t-test to examine if there was a statistically difference in the means of compliance rate before and after program implementation, and Fisher’s exact test to determine if the proportion of target compliance rate met was different before and after intervention. Results are presented in frequencies, percentages and means. No protected health information or patient identifiers were collected. The use of de-identified, publicly reported data in this quality improvement project limited the authors from conducting multivariate analyses factors associated with change in compliance rates before and after program implementation

## RESULTS

The two components of the project were implemented at the end of April 2021. For that reason, we did not include May 2021 in the analyses. The reported SEP-1 compliance measured by the compliance department and reported to CMS were compared prior to and post project implementation (Table 1).

**Table 1.** Pre-and Post-intervention SEP-1 bundle compliance rates.

	**Months pre-program implementation** **Nov 2020 to April 2021 (n=6 months) (n = 17)**						**Months after program implementation** **June 2021 to April 2022 (n=11 months)** **(n = 83)**											
	Nov20	Dec 20	Jan21	Feb21	Mar21	Apr21	Jun21	Jul 21	Aug21	Sep21	Oct21	Nov21	Dec21	Jan22	Feb22	Mar22	Apr22	p- value
Compliant Cases	4	2	3	4	2	2	10	7	10	10	5	7	6	10	7	6	5	
Total cases	6	2	1	6	4	8	13	13	12	14	9	12	10	15	12	13	9	
Compliance rate	67%	100%	33%	67%	50%	25%	77%	54%	83%	71%	56%	58%	60%	67%	58%	46%	56%	
Target rate	62%	62%	62%	62%	62%	62%	62%	62%	62%	62%	53%	53%	53%	53%	53%	53%	53%	
Target met (Yes/No)	Y	Y	N	Y	N	N	Y	N	Y	Y	Y	Y	Y	Y	Y	N	Y	
Average (SD) of percentages Pre vs Post^a^	0.57 (0.27); (95%CI: 0.29,.85)						0.62 (0.11); (95%CI: 0.55,0.70)											.562
Months in compliance^b^	3/6 months =50%						9/11 months = 81.8%											.280

^a^ Fisher’s exact test

^b^ t-test

For the five months prior to the program implementation, the average compliance rate was 57% (SD = 0.27; 95% CI: 0.29 - 0.85) in comparison with the 11 months post-program implementation period when the compliance rate was 62% (SD = 0.11; 95% CI: 0.55 - 0.70). Even when there was an increase of 5.4% from baseline, there were not statistically significant differences (p = 0.562) between the compliance rate before and after program implementation.

The institutional target percentage for SEP-1 compliance was 62%. The target percentage was decreased in October 2021 to 53%. The target percentage was met in 81.8% (9/11) of the months after program implementation in comparison with only 50% of the months (3/6) pre-implementation. During the post programming period, only one month was under 50% (46%) of compliance rate, that could disproportionately affect the overall compliance rate.

**Figure 3.**
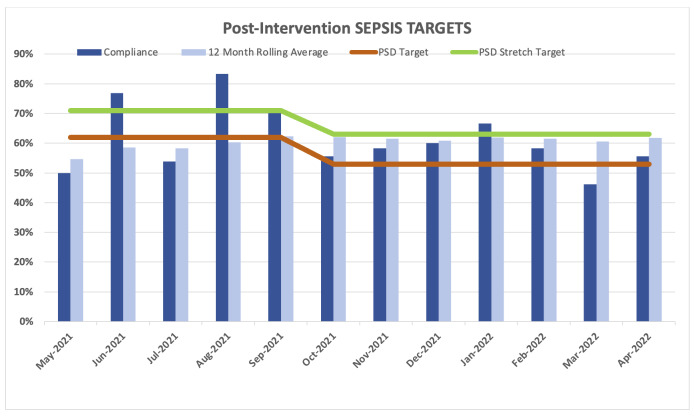
Post-intervention SEP-1 bundle compliance rates.

## DISCUSSION

After implementation of the Sepsis Macro and Sepsis Order Set, the overall compliance with the SEP-1 bundle improved from 57.0% to 62%. Although the project leaders were hoping for a larger improvement, the improvement did achieve compliance to the institutional target of 62%. The institutional target percentage for SEP-1 compliance was decreased to 53% in October 2021, which subsequently decreased the institutional stretch target rate to 63%. These institutional targets change each year after reassessment by the quality team to make it more possible for the hospitals to achieve a better score and improve the results and to strive for better scores from the previous year.

A comparison study using national data during 2017 fiscal year found a mean of 48.9% (SD = 19.4; range 0 to 100%) SEP-1 bundle compliance rate, reporting differences(n = 17 Pre, n = 83 Post-implementation) based on hospital-level characteristics. The study findings suggested that SEP-1 compliance was positively associated with ED-based process measures for time-sensitive care. This study also explained the variability of compliance among hospitals as an effect of the diversity of cases presented, with smaller, for-profit, non-teaching hospitals reporting higher SEP-1 bundle completion rates.^12^ Since the implementation of the Sepsis Macro and Sepsis Order Set, compliance rate at the authors’ teaching hospital sustained over 50% except for one month, higher than our previous compliance rates and over the 48.9% found at national level.^11^ This supports the theory that interventions in the ED, where both arms of the SEP-1 bundle originate, can improve hospital SEP-1 performance at a teaching hospital where compliance rates tend to be lower.

As part of the project, between three and five cases that were deemed noncompliant were reviewed by the ED providers each month during the emergency medicine operations meeting. Cases that were deemed noncompliant ‘fell out’ for multiple reasons. Frequencies of each reason for ‘falling-out’ were not measured. Cases chosen for review by the quality department were chosen if the reason for ‘falling-out’ were under the control of the emergency department providers. Frequently occurring reasons for ‘falling-out’ included: labs were not drawn and sent on time, antibiotics were not administered on time, and physicians’ failure to order vasopressors in a specified timeframe when indicated. The data used for evaluation of the project is the de-identified quality data that is extracted by the quality department for public reporting. Although charts that were non-compliant were identified by the quality team each month and reviewed by the EM physicians for continuous quality improvement, that data was not broken down into non-compliance types nor was reported. And due to turnover in the position, there was no standardization between data extractors to collect and report the non-compliance types. Thus, specific data for patients “falling out” are not available. Future PDSA cycles of the project can collect this data to better identify specific areas for “falling out”.

Because these components of the bundle were each directly addressed by the intervention, improvement in overall compliance to the bundle was likely also due to improvement in these areas. Other cases that ‘fell out’ included when a patient is acutely ill and quickly transferred from the ED to the inpatient ward, creating a gap in orders placed between the ED and the inpatient ward. Future interventions can incorporate order sets that can be continued once the patient is admitted to the inpatient ward.

The data analyzed is the de-identified data the institution reported to CMS. The reported SEP-1 compliance measured by the compliance department and reported to CMS were compared prior to and post project implementation. Results are presented in percentages. No protected health information or patient identifiers were collected. The de-identified, publicly reported data limited the authors from conducting more controlled analyses comparing monthly, pre- and post- implementation analytic procedures.

## CONCLUSIONS

This study demonstrated that a quality improvement project consisting of education on quality metrics and standardization in documentation and order entry in EMR’s by creating standard order sets and macros for providers to use can improve compliance to CMS HQI metrics in community-based teaching institutions.

### Conflict of Interest

None
